# Acceptability of a ‘guidebook’ for the management of Osteoarthritis: a qualitative study of patient and clinician’s perspectives

**DOI:** 10.1186/1471-2474-15-427

**Published:** 2014-12-13

**Authors:** Andrew Morden, Clare Jinks, Bie Nio Ong, Mark Porcheret, Krysia S Dziedzic

**Affiliations:** Research Institute for Primary Care & Health Sciences, Keele University, Keele, Staffordshire ST5 5BG UK

**Keywords:** Osteoarthritis, Self-management, Written information, Clinical guidelines, Qualitative, Health care professionals, Patients

## Abstract

**Background:**

Written information can be of benefit to *both* practitioners and patients and the provision of quality information is emphasised as a core intervention by United Kingdom National Institute of Clinical Excellence (NICE) OA guidelines. Researchers, patients and HCPs developed an ‘OA guidebook’ to provide; a) a balanced source of information for patients; b) a resource to aid practitioners when discussing self-management. This study aimed to evaluate the acceptability and usefulness of the OA guidebook as part of complex intervention to deliver NICE OA guidelines in General Practice.

**Methods:**

The intervention comprises a series of consultations with GPs and practice nurses in which supported self-management is offered to patients. Eight practices in the West Midlands and North West of England were recruited to take part: four control practices and four intervention practices. Semi-structured interviews were undertaken with patients (n = 29), GPs (n = 9) and practice nurses (n = 4) from the intervention practices to explore experiences of the intervention and use of the guidebook. Data were analysed using thematic analysis and constant comparison of data within and across interviews.

**Results:**

GPs thought the guidebook helped provide patients with information about OA aetiology, prognosis and self-management. Thus, it backed up key messages they provided patients during consultations. GPs also found the guidebook helped them ‘close off’ consultations. Nurses also thought the guidebook helped them describe OA disease processes in consultations. Patients valued the explanations of disease onset, process and prognosis. The use of ‘real’ people and ‘real life’ situations contained within the guidebook made self-management strategies seem more tangible. A sense of inclusion and comfort was obtained from knowing other people encountered similar problems and feelings.

**Conclusion:**

An OA specific written information guidebook was deemed acceptable and useful to practitioners and patients alike as part of the MOSAICS study. Findings reinforce the utility of this model of patient information as a resource to support patients living with chronic illnesses. An OA guidebook featuring a mixture of lay and professional information developed by professionals and lay people is useful and could effectively be used more widely in usual care.

**Electronic supplementary material:**

The online version of this article (doi:10.1186/1471-2474-15-427) contains supplementary material, which is available to authorized users.

## Background

Osteoarthritis (OA) related joint pain is highly prevalent and can cause disability, reduced quality of life and detrimental costs to individuals and society[1], and OA is one of the most common reasons for primary care consultations[2]. When patients consult for OA they frequently depart thinking that little can be done or that OA is an unimportant condition. This is because joint pain is often described as ‘wear and tear’ or related to ageing[3–5] and patients are rarely offered the full spectrum of treatments or self-management advice as recommended by clinical guidelines[4, 6, 7].

The provision of written information about health conditions is now a recognised strategy to facilitate patient centred shared decision making and self-management support[8–13]. To enhance shared decision making in the context of clinical guidelines, it has been suggested that such information needs to convey unbiased information on the pros and cons of treatment options including likely benefits and harms[14]. Self-management can be broadly defined as coping with difficulties, getting on with life and maintaining ‘self’ and relationships whilst living with a chronic condition. This features a complex process of using existing knowledge and resources whilst also utilising biomedical information and advice to manage symptoms as appropriate in a recursive manner[15–17].

One model of providing long term condition (LTC) self-management support is the Expert Patients Programme (EPP); a lay led, structured, course[18]. Kennedy and colleagues’ national evaluation of the EPP found that it was likely to be cost effective and increased self-efficacy and energy levels amongst patients, but highlighted three limitations. First, regards the ability to adequately provide disease specific information about individual LTCs. Second, relates to the EPPs capacity to respond to patient’s individual needs within social contexts. The final criticism pertains to the EPP obscuring the role of other models of care and eradicating clinical input into self-management support[17, 19, 20]. Consequently Kennedy and colleagues argue an approach which differentiates between LTCs and maximises input from healthcare professionals is a more appropriate model of self-management support in primary care (the WISE model)[17, 20]. The provision of condition specific written information is an important component of this model of care because it can potentially be of benefit to *both* primary care practitioners and patients when discussing supported self-management[17]. This is because written information can provide patients and Health Care Practitioners (HCPs) with a tangible detailed resource to anchor consultations and engage in collaborative decision making[11, 17].

Patient information provision is not a without its challenges. Some information sources do not meet the needs of patients because they are written with the assumption that patients are ill informed and passive[21, 22] and are orientated towards ensuring compliance rather than informing and empowering[10]. The content of information frequently does not relate to everyday life, actual behaviours and contexts and should reflect this because patient decision making and illness management is a complex, changeable, and longitudinal process set within social context(s)[10, 11, 23–25]. Other commonly identified patient information weaknesses include it being poorly presented and overly complex[10], not taking into account how patients interpret information[21, 26], failing to adequately incorporate lay and biomedical advice[21, 23, 27], and not offering hope or providing reassurance about the prognosis and effects of chronic long term conditions[23].

Responding to patient need is not the only consideration facing those who develop patient information. Qualitative research undertaken by Kennedy and Rogers demonstrated that written information is more likely to be valued by HCPs if it delivers clear biomedical messages that can help back up what they consider to be the core purpose of consultations, yet marrying tensions between ‘illness’ and ‘disease’ perspectives can present a challenge[11]. This is often compounded by inadequate involvement of all stakeholders in the development of materials[11, 24]. Kennedy and colleagues developed and tested patient information resources (or ‘guidebooks) for ulcerative colitis and irritable bowel syndrome. They recommend that patient information should include the latest scientific evidence and experiential knowledge about illness and presented in a user friendly manner which has been approved by patients and clinicians[10, 11, 23, 28].

Written information about OA has been critiqued for being contradictory, confusing, and solely providing biomedical ‘disease’ information rather than grounded in patients’ experiences of ‘illness’[24]. Booklets to improve OA self-management using patient centred messages have been trialled and ‘proof of principle’ established for future use[29–31]. Despite proof of principle, little is known about how useful written sources of information about OA are for HCPs and patients in everyday practice. The Medical Research Council’s (MRC) guidance on developing and evaluating complex interventions[32] recommends using evidence (such as clinical guidelines), engagement (with HCPs or patients, for example), and qualitative methods to not only develop components of interventions but to also to evaluate them[33, 34]. Therefore an ‘OA guidebook’ was developed using continuous input from patients and professionals to provide a balanced, user friendly information resource[26]. It was subsequently deployed as part of a complex intervention to improve the management of OA in general practice[35]. This article reports the HCPs’ and patients’ views on the acceptability and usefulness of the OA guidebook.

## Methods

### The OA guidebook

The development of a booklet entitled *A guide for people who have osteoarthritis* was led by researchers from Keele University in the United Kingdom and mirrored the MRC guidance for developing the components of interventions by combining evidence, engagement with stakeholders and qualitative research[32, 33]. A full description of the development of the guidebook is detailed elsewhere[26]. Briefly, for context, the development process featured a researcher undertaking a qualitative synthesis of patients experiences of OA from existing literature, sending findings (themes) separately to a lay panel and a professional panel convened to develop the content of the guidebook, and conducting a meeting interview with each group to identify information needs. The researcher then composed an initial draft of the guidebook with the assistance of a reference group (whose remit was to ensure the most up to date clinical information was included) before sending the first draft to the lay and professionals panels for comment (collated via individual or group interviews). Subsequently the guidebook was redrafted (with assistance from the reference group) and was sent to the lay and professional panels for further comment, again collated by group or individual interviews. A third iteration was drafted in tandem with the reference group before being finalised. The guidebook contains the following chapters: 1) Personal experiences of joint pain, 2) Understanding joint pain as a diagnosis, 3) Seeking professional help, 4) Managing and treating joint pain (essentials), 5) Managing and treating joint pain (adding to the essentials), 6 ) Feeling positive, as well as a list of support organisations. An online version of the guidebook can be accessed here: http://www.arthritisresearchuk.org/arthritis-information/keele-oa-guide.aspx.

### Context of use

**Figure 1:**
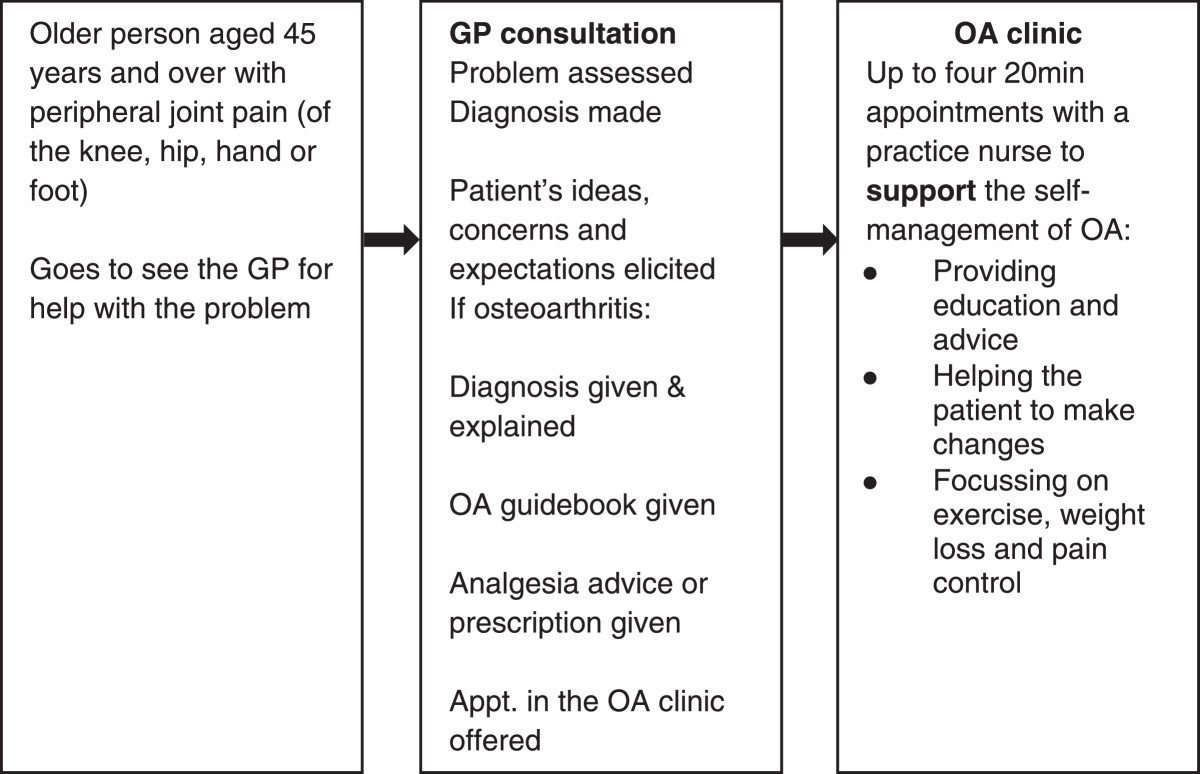
**MOSAICS trial intervention.**

The guidebook was used as part of the *Management of Osteoarthritis in Consultations Study: the development of a complex intervention in primary care (MOSAICS)*, a cluster randomized control trial. Full details of the trial are available from the study protocol[35]. The trial aimed to enhance the supported self-management provided to patients and promoted the uptake of the core treatments recommended in NICE OA guidance[12]. The intervention consisted of a semi-structured GP consultation, use of the OA Guidebook[26] and referral to a nurse-led OA clinic to provide up to four appointments to support self-management. The intervention was designed so that the guidebook could support consultations and be used by patients as a stand-alone resource, based on the WISE model, which emphasizes the provision of appropriate information for patients (in the form of a guidebook), professionals being responsive to patient need and context, and services that are readily available to patients[17]. The intervention is detailed in Figure 1.

Eight practices in the West Midlands and North West of England were recruited to take part in the study: four control practices and four intervention practices. The trial featured a series of sub-studies to evaluate the intervention. This paper reports on HCP and patient views on the use of a guidebook as part of the primary care based intervention. Ethical approval to implement and undertake all research components of the intervention was obtained from the local NHS research ethics committee (ref:10/H1017/76).

### Study design

A qualitative design was used in this sub-study. In-depth interviews were chosen because they yield rich exploratory data[36] and here offered the opportunity to understand patients’ perspectives about the appropriateness of information offered, and if and how understanding of OA and its management was influenced by the guidebook. A qualitative methodology was appropriate for understanding HCPs’ views and perspectives of the guidebook in relation to benefits to patients and their own clinical practice. The respondent could convey their experiences in their own words, illuminating the meaning that they attached to events or how the guidebook was used in social settings[37].

#### Sample selection and recruitment

A ‘criterion-i’ (p5) sampling strategy was used[38], an approach which emphasises recruiting individuals who have detailed experience and knowledge of a phenomenon or event(s) in a given context[38]. In this case, practitioners who had delivered the intervention and patients who had consulted and received the guidebook. All GPs and nurses from the four intervention practices were invited to take part in interviews (either face to face or via telephone) after the trial had been completed. They were asked about delivering the intervention, their thoughts about the guidebook and how they used it in clinical practice. The practices were a mix of rural and urban and varied in size (in terms of patient populace). GPs and Nurses were sent an invitation letter and information sheet. Nine GPs and four practice nurses from intervention practices agreed to be interviewed.

During the trial, all patients who had consulted for OA at the intervention practices and consented to further contact were eligible to participate in the qualitative study. All participants in the trial were sent baseline, 3 month and 6 month ‘consultation questionnaires’. Those who indicated they had seen the GP and nurse for OA in the last 3 months were sent an invitation letter and information sheets about the qualitative study. Of the 49 invited to take part in interview, 29 agreed to do so.

The sample size was based on participants who agreed to take part as opposed to ceasing once data saturation was reached. Participants were all white British, 19 were female, and 10 were male. The youngest participant was 45 and the oldest was 72.

#### Data collection

An initial interview topic guide was developed by drawing from existing literature and developed further after discussion amongst the study team. The interview guide was refined to reflect themes and topics that emerged from continuous data collection and analysis (see below)[39]. Interviews with HCPs were either undertaken face to face at their practice (n = 2) or via telephone (n = 11) between March and June 2013 by AM, CJ and BNO. All patient interviews were undertaken in participants’ homes by the lead author between May 2012 and May 2013. All participants provided informed written consent prior to interviews commencing.

#### Data analysis

All interviews were audio recorded and professionally transcribed verbatim. Whilst a full Grounded Theory[39] approach was not adopted, key principles of the Grounded Theory analysis process were followed; first, the lead author independently read and closely coded transcripts. Initial coding of 8 transcripts was consolidated into themes. This process was strengthened by BNO, CJ and KSD undertaking separate analysis, followed by team discussions to arrive at agreement regarding coding and interpretation[40]. The lead author then continued coding subsequent interviews. Any additional codes that emerged from ensuing interviews were then incorporated into the coding scheme and all preceding transcripts re-checked. Memos were used during analysis to record developments in coding and make connections between themes[39].

## Results

Themes that emerged from analysis of each set of interviews are presented below. Findings from HCP interviews are detailed first before findings from patient interviews are reported.

### HCP interviews

#### HCPs perceptions of benefits to patients

The guidebook was seen by HCPs as an appropriate aide memoire that patients could turn to when they needed information as and when required. This in particular was aided by the guidebook featuring a clear format and language which avoided clinical terminology or jargon. First, the GPs and nurses suggested that the guidebook clarified perceived patient confusion between osteoarthritis and rheumatoid arthritis and clarified what kind of *disease* they have.

A second reason for the guidebook’s benefit that HCPs suggested was because it provided patients with a description of causality and prognosis:

*I think that really helps with patients understanding what arthritis is, that it isn’t a disease some people catch and some people don’t catch and that just because your mother has it you might get it, I think people understand the process more that’s it’s a dynamic process it’s not just a one way slope (GP6).*

As the example highlights, HCPs argued that patients benefited from biomedical information about the aetiology of OA, insofar it clarifies ‘lay’ understandings of OA causation and suggests that OA is not necessarily debilitating.

HCPs thought a key strength of the guidebook was that it provided a description of the underlying disease process of OA:

*So I think improving patients understanding of what’s happening with the kind of flare and repair idea makes them understand that they can reduce their pain during a flare so that their body’s constantly trying to repair and that they can encourage that (GP2).*

Providing correct information about ‘disease’ was argued to be what patients would take from the guidebook. Whilst HCPs framed these issues as being beneficial to patients, arguably they highlighted the things directly related to their practice.

#### Managing consultations

The benefits that patients were thought to gain from the guidebook coincided with how GPs thought that the guidebook helped them to back up the key messages they delivered regarding the treatment and management of OA:

*It’s always good to be able to sort of back up what you’re saying to a patient with something written, I’m never sure how much of the book they actually do read but those who are engaged hopefully will take that opportunity (GP7).*

Whilst the GP expresses some uncertainty regarding the likelihood of all patients reading the guidebook, she outlines its usefulness for highlighting key topics in the guidebook during consultations and encouraging patients to focus on them.

GPs also thought that consultations could ‘empower’ patients to look after their own condition. The guidebook was depicted as a tool to help this process:

*‘Your book, your thing, I want you to read it all. I want you to bring any questions.’ Making them in charge of their health, responsibility of their problem, engaging them, almost, and I hate this word, but empowering them (GP4).*

The guidebook was a symbolically useful object, conveying to the patient that by giving them their personal booklet they were expected to be responsible for their condition.

Furthermore, GPs contended that when they handed out the guidebook it helped bring consultations to a more comfortable conclusion:

*I always felt talking about things like the footwear and exercise and weight loss that patients are just a bit weary of it and go away sometimes thinking “you know my doctor’s not done anything for me” but to actually give them that backed up with written information I really think that makes a difference to the impact (GP8).*

GPs interpreted providing something tangible containing condition specific information was acceptable for patients because it meant they had gained at least one thing from the consultation. In turn this, in part, symbolised a successful, well managed, consultation for GPs because it had been neatly ended. Thus, for GPs, the guidebook provided a resource with which to back up key messages about self-management, an aid to empowering patients and for ending consultations smoothly.

Nurses also suggested that the guidebook helped aid consultations. They emphasised that the guidebook helped explain the disease process of OA and how it affected patients from the *outset:*

*I would say in the first consultation probably just as a way of helping to explain what OA was (Nurse2).*

Thus, for nurses the guidebook as a consultation resource helped to ‘set the scene’ by providing a description of the condition to patients and it formed a launch pad for subsequent care they provided.

Nurses, like the GPs, found it a useful resource for reiterating the key messages about self-management (for example keeping active or taking medication) that they delivered as part of the intervention:

*The guide book was good because the doctors gave those out and they’d read a little bit about what we were going to do prior to coming to see me so all I did was sort of reinforce the information so that was good (Nurse3).*

The guidebook helped reinforce key messages, but unlike GPs nurses focussed less on the ‘empowering’ utility of the guidebook. Whilst nurses positioned the guidebook as useful for reinforcing basic messages about ‘keeping active’ and ‘exercising’ they used supplementary sheets produced by Arthritis Research UK to demonstrate particular muscle strengthening exercises. This was because they thought that the guidebook lacked specific information about muscle strengthening exercises they were encouraged to discuss with patients as part of the intervention training. In summary, for nurses the guidebook played less of a role in aiding consultations and mainly featured at the outset or as an occasional tool to bolster the advice they sought to convey.

### Patient interviews

#### Clarifying and explaining OA

The general sentiment expressed by participants was encapsulated by one participant who stated “I think it was well written and easy to understand”. The majority of patients outlined how previously they had not obtained consistent or digestible written information and this was compounded by not being given a detailed description of OA by GPs. Thus, participants suggested that the guidebook helped to make up for the shortfall and provided an explanation about what OA is. Patients outlined two ways in which OA was explained and clarified. First, they discussed how the guidebook explained the onset of OA and the underlying disease process:

*I found it very interesting and it gave me a different insight. Okay, yes, I had a medical background, but somehow it’s different when you have it yourself. And I think it sunk in more, and it’s not always hereditary, it can be trauma, it can be all sorts of things, weight, etc. And just seeing the joints and how it affects the joints I found very helpful and very interesting (Patient26).*

This participant had worked as nurse before her retirement. She outlines how despite her ‘medical’ training she was unsure about OA’s mechanics and causal factors, and the guidebook was useful because it related to her current situation as a patient.

Second, patients appreciated that the guidebook contained a *prognosis.* Again, they interpreted that the information exceeded what they were offered by GPs:

*I think one thing that stood out was that the condition can get better; it doesn’t have to get worse. Initially my wife was told that in her case the cartilage is worn and that’s it basically, she’s never going to get it back. So therefore you can soldier on for a while but the pain’s going to get worse and the mobility is going to get worse and in the end you’re going to have to have a knee replacement. But now it seems that, for whatever reason, people are now suggesting that it can replace itself or regenerate itself or it can rebuild in some way. So that’s encouraging (Patient18).*

This participant was not given a prognosis and also invokes the experience of his wife who had interpreted the advice given in a previous consultation as meaning her future inevitably featured disability and operations. He compares the experience of his wife with the guidebook which explains that OA is part of a process of *repair* rather than degeneration. He has gained a sense of optimism and reassurance because it offers a more positive outlook to life with OA.

Other participants knew they had a form of ‘arthritis’ , but were not quite sure which type. They were relieved to find out that OA was not likely to lead to incapacity:

*I’m glad to know that I haven’t got the one that… rheumatoid. You get one and it will cripple you and you get another one that you can actually live with. So I was quite pleased when I found out the information out of the book (Patient17).*

As this quote exemplifies, the guidebook offered clarification about disease type, and reassurance that OA was not equated to an inexorable decline and a reduction in quality of life.

In summary, the guidebook provided a more detailed explanation of why patients had pain, its cause and the likely future outcome of OA, which offered a sense of reassurance.

#### Providing a sense of inclusion

The positive aspects of the guidebook patients discussed transcended the provision of biomedical information because it provided them with a sense they were not alone:

*This bit about where it tells you about people with this, saying the symptoms, I thought that was quite good you know it makes, you know, it makes you feel that you’re not on your own don’t it (Patient4).*

As this participant exemplifies, reading about symptoms and concerns being common and normal makes the problem seem less of a personal burden and unique worry.

Other patients emphasised the value of the guidebook containing quotes about emotional experiences of others. This woman spoke about the frustration she felt at pain limiting her activities:

*I mean there was quotes and in reading them it’s sort of, yes I feel like that sometimes and you know, so it’s almost as though you’re not alone (Patient23).*

By reading about issues as stated by other ‘regular’ people who felt similarly frustrated she gained a sense of inclusion and she felt reassured that her thoughts and feelings were reasonable, common and natural.

A final way that the guidebook promoted a sense of inclusion related providing details of groups or health-care professionals to reach out to in order to avoid isolation and obtain advice. For participants, the guidebook helped ease anxiety by signposting the way to getting appropriate help, support or advice.

#### Aiding self-management

Participants already engaged in some form of self-management, either self-learned from experience or disseminated via social networks. They had also received advice on self-management during consultations with nurses and they discerned a clear role for the guidebook. First, participants used it in tandem with advice from nurses for reassurance that existing activities or hobbies were helpful or not harmful:

*It confirms what you’re suspecting yourself, but you’re not sure. It just inspires you to say, ‘right, well, I will carry on doing this, this obviously seems to be the way forward’ (Patient29).*

This participant was pleased to find that she could continue existing physical activities without worry about damaging joints or causing additional pain. Other participants focused on how the guidebook reassured them about how they used painkillers or supplements (and reinforced the advice provided by nurses on this topic).

Second, participants gained information about what they did not know, or what had not been discussed during consultations with the nurse such as using TENS machines, or aids and devices.

Third, patients used the guidebook as a counterbalance to things they had discussed with the nurse but had not felt entirely comfortable with:

*The bits I found most interesting and probably the most helpful, are the bits about exercise, about keeping mobile, keeping moving. Finding something you enjoy doing, because there’s a lot of stuff I don’t like doing; I hate sports (Patient28).*

Being advised to find an activity that suited their preferences was not only practical advice, but delivered in a way that made sense and was acceptable. It also counterposed the nurse’s focus who concentred on muscle strengthening exercises and aerobic fitness, which was not always something patients wanted to do.

In summary, the guidebook provided a flexible tool with which to support individuals’ preferred self-management approaches, which may or may not directly mirror professional advice.

#### Patient criticisms of the guidebook

The guidebook was not without criticism from patients. Whilst all patients found an element of the guidebook useful, some described misgivings about the relevance, usefulness and presentation of some of the material. Some (n = 2) participants suggested that the guidebook did not resonate with their personal situation. Both participants were in their late 40s and thought that the images of older adults engaged in activities did not relate to their identities as ‘young’ people.

A complaint from a small group of participants (n = 3) was that the guidebook duplicated lifestyle messages they had already obtained because of co-morbidities. One man commented that “in the booklet there’s a lot on diet and then the Stroke Association stuff, again, there was a lot on diet, and they overlap”. For him it felt like another indistinguishable set of materials about lifestyle. However, these participants did at the same time appreciate other aspects of the guidebook such as the explanation of OA and its prognosis.

One participant asserted that the guidebook adopted a patronising tone: “Some of it (long pause) I found was (long pause) a bit like you were talking to a child. I like to think I’ve got quite a bit of common sense, so it seemed a bit irrelevant”. This participant acknowledged that for other people the guidebook would be helpful and was the only individual to raise this issue.

## Discussion

HCPs found written information about OA developed by patients, academics and clinicians useful. The guidebook helped to deliver key medical messages and aided consultations[11]. GPs and nurses positioned the guidebook as beneficial to patients because it provided a medical explanation about OA and corrected lay beliefs about illness. One interpretation is that they focused on what they thought *should* be of use to patients.

GPs thought that the guidebook backed up their advice, empowered patients, and encouraged them to take ownership of their condition. Nurses valued the guidebook because it helped them convey biomedical knowledge to patients, bolster key messages about self-management, and helped to ensure patients adhered to advice. Nurses drew attention to the lack of content about specific muscle strengthening exercises, which they saw as a key to their role in providing care.

There was some congruence between what HCPs and patients liked about the guidebook. Patients appreciated biomedical information because it filled a void in their knowledge. It explicated the onset of OA as a disease process, and its prognosis. Patients noted that the guidebook provided information and explanation that was lacking from consultations with their GP. The guidebook also helped patients live with ‘illness’ because it provided a sense of inclusion, reassured them what they were already doing was correct (in tandem with nurse consultations), offered new treatment options not previously provided by usual care, and helped patients negotiate professional advice they did not necessarily appreciate[11]. A small number of patients voiced criticism about certain information or the presentation of the guidebook. However, these patients simultaneously found something useful within the guidebook and on the whole it was valued by participants.

Whilst insight into the precise content of consultations cannot be obtained via interviews, the data suggests that some GPs were not providing clear ‘disease’ information to patients and were happy to let the guidebook fulfil this role. Using the guidebook in this way could have a potentially detrimental effect especially since patients value the expertise and validation that GPs provide[41]. There was dissonance between what patients and clinicians saw as the important focus of written information[11]. Grime & Pollock have emphasised the importance of constructing patient information with the needs of the patients in mind, not what HCPs think patients will need[21], whereas others recognise that HCPs should feel comfortable using patient information[11], thus a balance needs to be maintained. Care must be taken to balance the needs of HCPs and patients whilst avoiding the scenario where patient information becomes a substitute rather than a supplement to information provided in the GP consultation.

The study has limitations that need to be acknowledged. First, as discussed earlier, the sample of patients were all white British so no insights can be gained into what individuals from other ethnic groups would think of the guidebook, especially in relation to language and culture[42]. In addition the use of the guidebook was restricted to four practices who participated in a trial which means the findings may not be more widely generalizable to routine care. However, the findings do provide insights into what was helpful for these participants in this context[40], suggest what might be useful for patients and clinicians when developing written information, and indicate that additional testing of this guidebook is desirable.

## Conclusions

On the whole, the guidebook was an acceptable and useful resource for HCPs and patients who participated in this study, finely balancing the different perspectives that each group draw upon and find useful. The findings provide initial evidence that an OA guidebook featuring a mixture of lay and professional information developed by professionals and lay people is useful and could potentially be used more widely in usual care. Findings from this study reinforce the utility of this model of patient information as more broadly supportive to patients living with chronic illnesses[28, 30], but additional research with a larger sample or using mixed methods research is needed to thoroughly test the efficacy of the OA guidebook discussed in this study. The findings from this study also highlight some tensions and difficulties in creating written information which is sensitive and beneficial to patients whilst also satisfying the professionals who will be using the materials.

## Authors’ original submitted files for images

Below are the links to the authors’ original submitted files for images. Authors’ original file for figure 1
