# Supplementary material for: Acceptability of a ‘guidebook’ for the management of Osteoarthritis: a qualitative study of patient and clinician’s perspectives
Source: BMC Musculoskelet Disord. 2014 Dec 13;15:427. doi: 10.1186/1471-2474-15-427 (PMC4301067; doi:10.1186/1471-2474-15-427)
Supplement: Supplementary file 1 — Authors’ original file for figure 1 [file 12891_2014_2376_MOESM1_ESM.pdf]

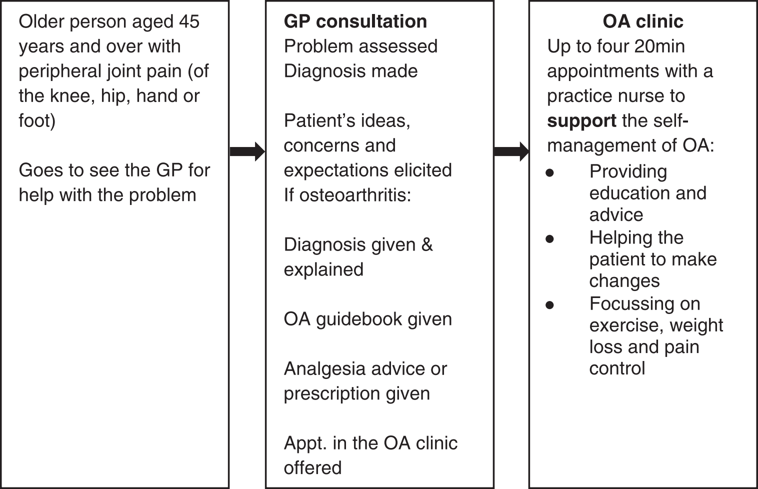
Older person aged 45 years and over with peripheral joint pain (of the knee, hip, hand or foot)
Goes to see the GP for help with the problem
GP consultation
Problem assessed
Diagnosis made
Patient's ideas, concerns and expectations elicited
If osteoarthritis:
Diagnosis given & explained
OA guidebook given
Analgesia advice or prescription given
Appt. in the OA clinic offered
OA clinic
Up to four 20min appointments with a practice nurse to support the self-management of OA:
Providing education and advice
Helping the patient to make changes
Focussing on exercise, weight loss and pain control
